# Azithromycin attenuates wheezing after pulmonary inflammation through inhibiting histone H3K27me3 hypermethylation mediated by EZH2

**DOI:** 10.1186/s13148-023-01430-y

**Published:** 2023-01-23

**Authors:** Shuqi Wu, Xiaochun Tian, Qian Mao, Chang Peng

**Affiliations:** Department of Pediatrics, Guizhou Children’s Hospital, Affiliated Hospital of Zunyi Medical University, 149 Dalian Street, Zunyi, 563000 Guizhou People’s Republic of China

**Keywords:** Wheezing diseases, Histone methylation, Azithromycin, EZH2, Pulmonary inflammation

## Abstract

**Background:**

Histone methylation modification plays an irreplaceable role in the wheezing diseases. The aim of this study was to explore whether azithromycin (AZM) attenuates post-inflammatory wheezing through inhibiting hypermethylation of histone H3K27me3 mediated by EZH2.

**Results:**

A randomized controlled trial was conducted on 227 children who underwent fiber-optic bronchoscopy, and bronchoalveolar lavage fluid (BALF) was collected for analyses. The expressions of IL-6, IL-2, NF-κB P65, EZH2 and H3K27me3 in the BALF of wheezing cases were significantly increased when compared with levels in non-wheezing cases (*P* < 0.05), while IL-10 was decreased (*P* < 0.05). AZM attenuated the overexpression of NF-κB P65, EZH2 and H3K27me3 in wheezing cases (*P* < 0.05) and shortened the time of wheezing in wheezing cases (*P* < 0.05). An in vitro model of inflammation was established using rat alveolar macrophages induced by lipopolysaccharide (LPS). AZM, SN50 (a NK-κB inhibitor) and GSK126 (an EZH2 inhibitor) attenuated the overexpression of EZH2, NF-κB P65 and H3K27me3 induced by LPS in rat alveolar macrophages (*P* < 0.05). AZM, SN50 and GSK126 normalized the decreased expression of IL-10 induced by LPS in the same samples (*P* < 0.05). Co-immunoprecipitation results showed that H3K27me3 interacted with EZH2 and NF-κB P65, and immunofluorescence data showed that AZM and SN50 inhibited LPS-induced NF-κB P65 nuclear translocation in rat alveolar macrophages.

**Conclusion:**

Histone H3K27me3 hypermethylation mediated by EZH2 may be involved in wheezing after pulmonary inflammation. AZM attenuated wheezing after pulmonary inflammation by inhibiting NF-κB P65-related hypermethylation of H3K27me3 mediated by EZH2.

## Background

The incidence of wheezing diseases in children has been increasing in recent years [1]. Prolonged wheezing can develop into asthma, which affects the quality of children’s life [2]. Increasing evidence has demonstrated that multiple factors can lead to wheezing in children, such as airway infection, allergy, foreign body in the airway and sleep apnea syndrome; however, airway infections are still the main cause of wheezing in children [3]. Some studies found that after airway infection, alveolar macrophages rapidly gather at inflammatory sites, resulting in an imbalance of secreted pro-inflammatory factors and anti-inflammatory factors in the lungs [4, 5]. However, the potential role of inflammatory cytokines in wheezing after pulmonary inflammation remains unclear.

Studies have shown that complex gene–immune environment interactions contribute to the development of wheezing diseases [6]. Histone methylation modification is an epigenetic mechanism that can regulate gene expression [7]. Histone methyltransferase EZH2, which specifically catalyzes the trimethylation of lysine 27 on histone H3 (H3K27me3) [8, 9], has been shown to be closely related to inflammatory responses and macrophage activation and asthmatic diseases [10–12].

NF-κB is a classical nuclear transcription factor. Many studies have demonstrated that NF-κB participates in various inflammatory responses through regulating target genes and mediating the expression of inflammatory cytokines [13]. One study found that activation of NF-κB-related signaling pathways promotes wheezing after respiratory syncytial virus infection [14], and inhibition of NF-kB p65 activation reduced airway inflammation in asthmatic mice [15]. Importantly, the activation of NF-κB-mediated inflammation-related signaling pathways is closely related to the regulation of histone H3 methylation [16–19]. However, whether NF-κB p65 is involved in the development of post-inflammation wheezing through regulating histone methylation modification is unknown.

Azithromycin (AZM) is a 15-membered macrolide antibiotic with antibacterial effects on many bacterial types, especially mycoplasma (MP). Recently, some studies found that AZM also shows non-specific anti-inflammatory activity and immunomodulatory effects in airway inflammatory diseases [20–22]. In addition, numerous studies and clinical observations showed that AZM alleviates wheezing symptoms in children and even delays asthma exacerbation [23, 24]. Furthermore, AZM significantly reduces airway inflammation, mucus secretion, airway remodeling and epithelial cell apoptosis induced by ovalbumin in mice [25, 26]. However, the mechanism of AZM in attenuating wheezing symptoms remains unclear.

Therefore, in this study, we explored whether EZH2-mediated histone H3K27me3 hypermethylation may be involved in wheezing after pulmonary inflammation and investigated the potential mechanism by which AZM attenuates wheezing after pulmonary inflammation. These findings may help identify novel strategies for clinical prevention and treatment of wheezing after pulmonary inflammation.

## Results

### Characteristics of clinical cases

This study included data from 227 children diagnosed with severe pneumonia with or without wheezing, bronchial foreign bodies, congenital laryngeal cartilage dysplasia or airway malformations, including 115 males and 89 females. There were 72 patients in the Non-wheeze group, 76 in the Wheeze + Vehicle group, and 56 in the Wheeze + AZM group; the median patient age was 5.2 (1–12, 2.95 ± 2.54), 2.6 (0.6–9, 2.05 ± 1.78) and 2.3 (0.6–8, 1.85 ± 1.55) years old, respectively. The control group included 23 participants, including 14 males and 9 females, with a median age of 2.9 (0.6–8, 2.60 ± 2.06) years old. There were no significant differences in age (χ^2^ = -− 2.464, *P* = 0.482) or sex (χ^2^ = -− 1.447, *P* = 0.694) between groups (Table 1).

**Table 1:** Comparison of general data between four groups [case (%)]

	Control group	Non-wheeze group	Wheeze + vehicle group	Wheeze + AZM group	*χ*^2^	*p*
	(*n* = 23)	(*n* = 72)	(*n* = 76)	(*n* = 56)		
Female	9 (39)	35 (48)	30 (39)	24 (43)		
Male	14 (61)	37 (52)	46 (61)	32 (57)	1.447	0.694
≤ 2.5 (Y)	11 (48)	36 (50)	42 (55)	35 (62)		
> 2.5 (Y)	12 (52)	36 (50)	34 (45)	21 (38)	2.464	0.482

*n*: sample size; *Y*: years; *p* value: significance level (probability of obtaining test results as extreme as the results observed)

### The expression levels of cytokines in bronchoalveolar lavage fluid (BALF) and plasma in children with wheezing after pulmonary inflammation

**Fig. 1:**
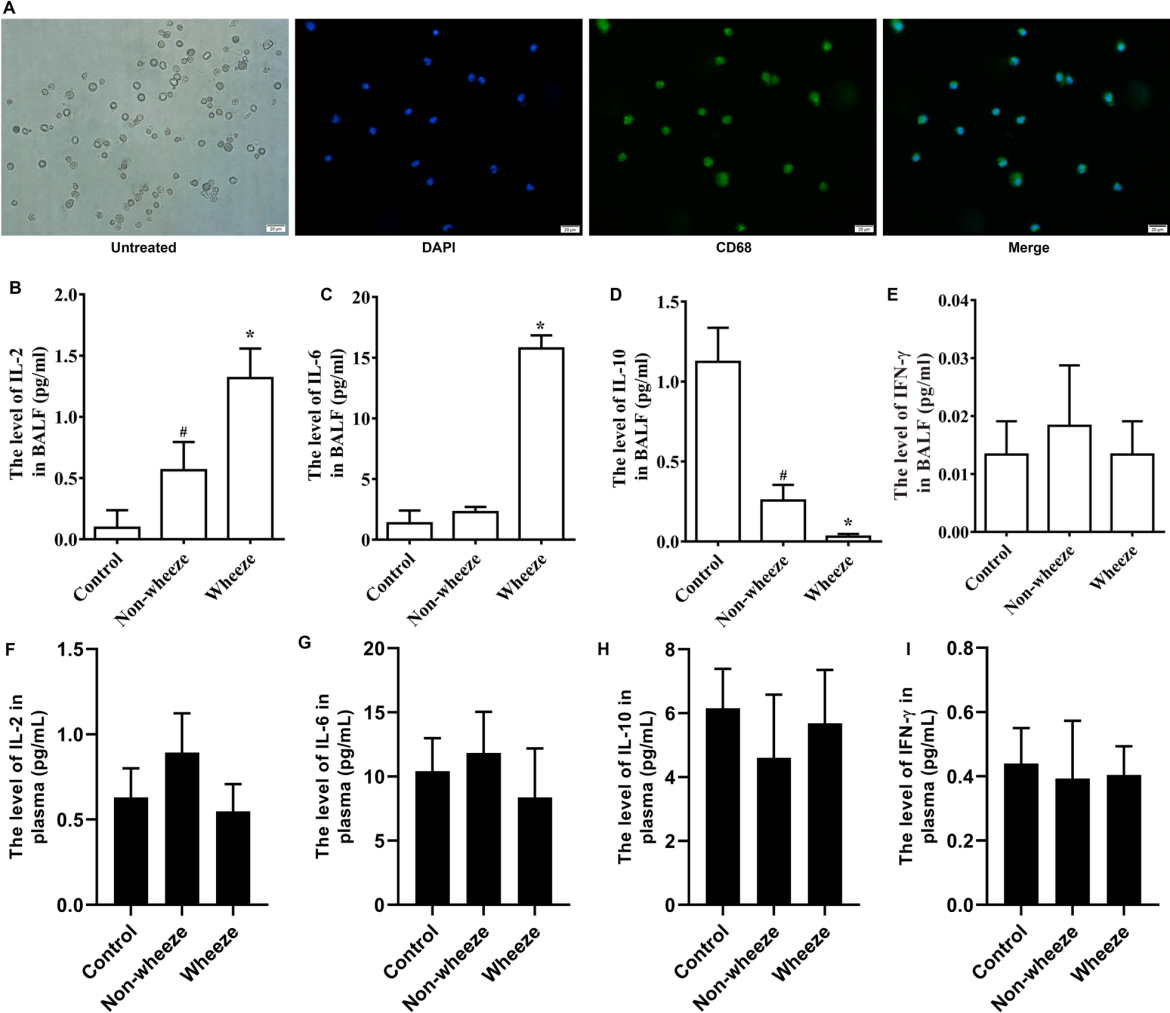
Expression levels of cytokines in BALF and plasma in patients with wheezing after pulmonary inflammation. **A** Identification of alveolar macrophages. The cells adhered to the wall and were round. Green (CD68), blue (DAPI). Scale bars indicate 20 μm. **B**–**E** The expression levels of IL-2, IL-6, IL-10 and IFN-γ in the BALF of the Non-wheeze group and Wheeze group. **F**–**I** The expression levels of IL-2, IL-6, IL-10 and IFN-γ in plasma of the Non-wheeze group and Wheeze group. *P < 0.05 versus the Control group and Non-wheeze group. ^#^*P* < 0.05 versus the Control group (*n* = 4)

BALF was collected from each group, and macrophages in BALF were identified by immunofluorescence staining for the macrophage-specific antigen CD68 (Fig. 1A). ELISA of BALF supernatant showed that the expression levels of cytokines IL-2 and IL-6 in the Wheeze group were higher than those in the Control group and Non-wheeze group (*P* < 0.05; Fig. 1B, C), while the expression level of cytokine IL-10 in the Wheeze group was lower than that in the Control group and Non-wheeze group (*P* < 0.05; Fig. 1D). IFN-γ levels showed no differences among groups (Fig. 1E). To clarify whether cytokines in BALF or plasma played a role in wheeze after pulmonary inflammation, we examined cytokines in the plasma of the same cases by ELISA. The results showed no significant change in the expression levels of IL-2, IL-6, IL-10 and IFN-γ in plasma among groups (Fig. 1F–I). Together, these data suggested that an imbalance of cytokines in BALF may be involved in wheezing induced by pulmonary inflammation.

### Histone H3K27me3 hypermethylation and EZH2 overexpression in the BALF in children with wheezing after pulmonary inflammation

**Fig. 2:**
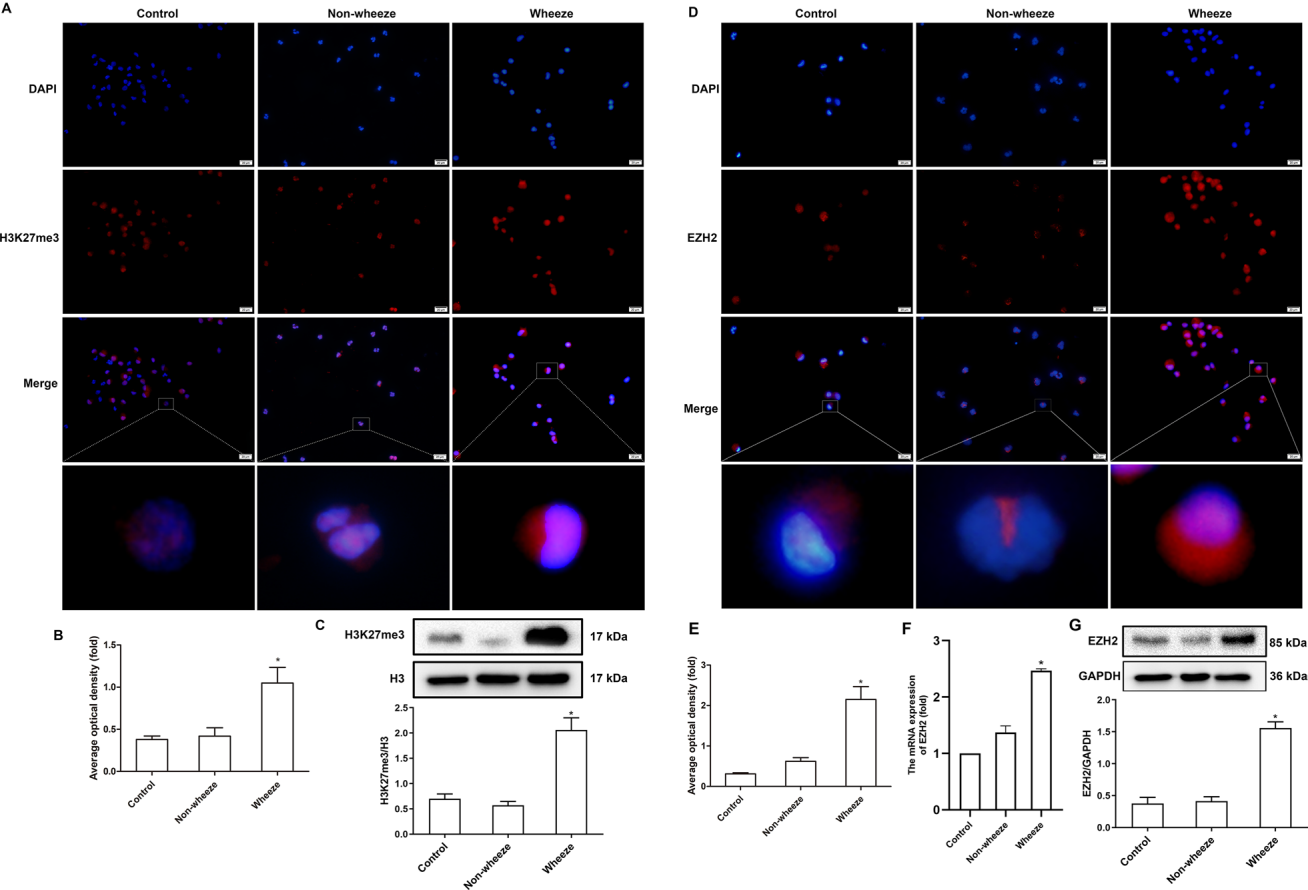
Expression levels of H3K27me3 and EZH2 in BALF of patients with wheezing after pulmonary inflammation. **A** Histone H3K27me3 in the BALF was detected by immunofluorescence staining. Red (H3K27me3), blue (DAPI). The scale bars indicate 20 μm. **B** Average optical density of H3K27me3 immunofluorescence in the three groups (*n* = 3). **C** The expression level of histone H3K27me3 in the BALF was detected by Western blotting (*n* = 10). **D** The expression level of EZH2 in the sediment of BALF was detected by immunofluorescence staining. Red (EZH2), blue (DAPI). The scale bars indicate 20 μm. **E** Average optical density of EZH2 immunofluorescence in the three groups (*n* = 3). **F** The mRNA expression of EZH2 in the BALF was detected by RT-qPCR (*n* = 10). **G** The expression level of EZH2 in the BALF was detected by Western blotting (*n* = 10). *P < 0.05 versus the Control group and Non-wheeze group

Histone methylation plays a critical role in the development of infectious diseases and wheezing disease [10, 11]. We examined histone H3K27me3 in the BALF by immunofluorescence staining and western blotting. Immunofluorescence staining showed that histone H3K27me3 methylation was significantly increased in the Wheeze group compared with the levels in the Control group and the Non-wheeze group (*P* < 0.05; Fig. 2A, B). Western blotting results also showed that the expression level of histone H3K27me3 in the Wheeze group was higher than that in the Control group and the Non-wheeze group (*P* < 0.05; Fig. 2C). These results suggested that an increase in histone H3K27me3 methylation may be associated with wheezing induced by pulmonary inflammation.

EZH2, a subtype of histone methyltransferases, plays an important role in transcriptional repression by catalyzing histone H3K27me3 methylation to compact chromatin, which closely related to wheezing diseases [8, 9]. Immunofluorescence staining results showed that compared with the Control group and the Non-wheeze group, the Wheeze group showed significantly increased expression levels of EZH2 (*P* < 0.05; Fig. 2D, E). The data of RT-qPCR and Western blotting also showed that the mRNA and protein expression levels of EZH2 in the Wheeze group was increased significantly compared with the levels in the Control group and the Non-wheeze group (*P* < 0.05; Fig. 2F, G). There was no significant difference in EZH2 levels between the Control group and the Non-wheeze group. These results implied that EZH2-mediated histone H3K27me3 methylation imbalance may be involved in the occurrence of wheezing after pulmonary inflammation.

### AZM attenuated the induced expression of NF-κB P65, EZH2, H3K27me3 and cytokines and shortened wheezing time in wheezing children

**Fig. 3:**
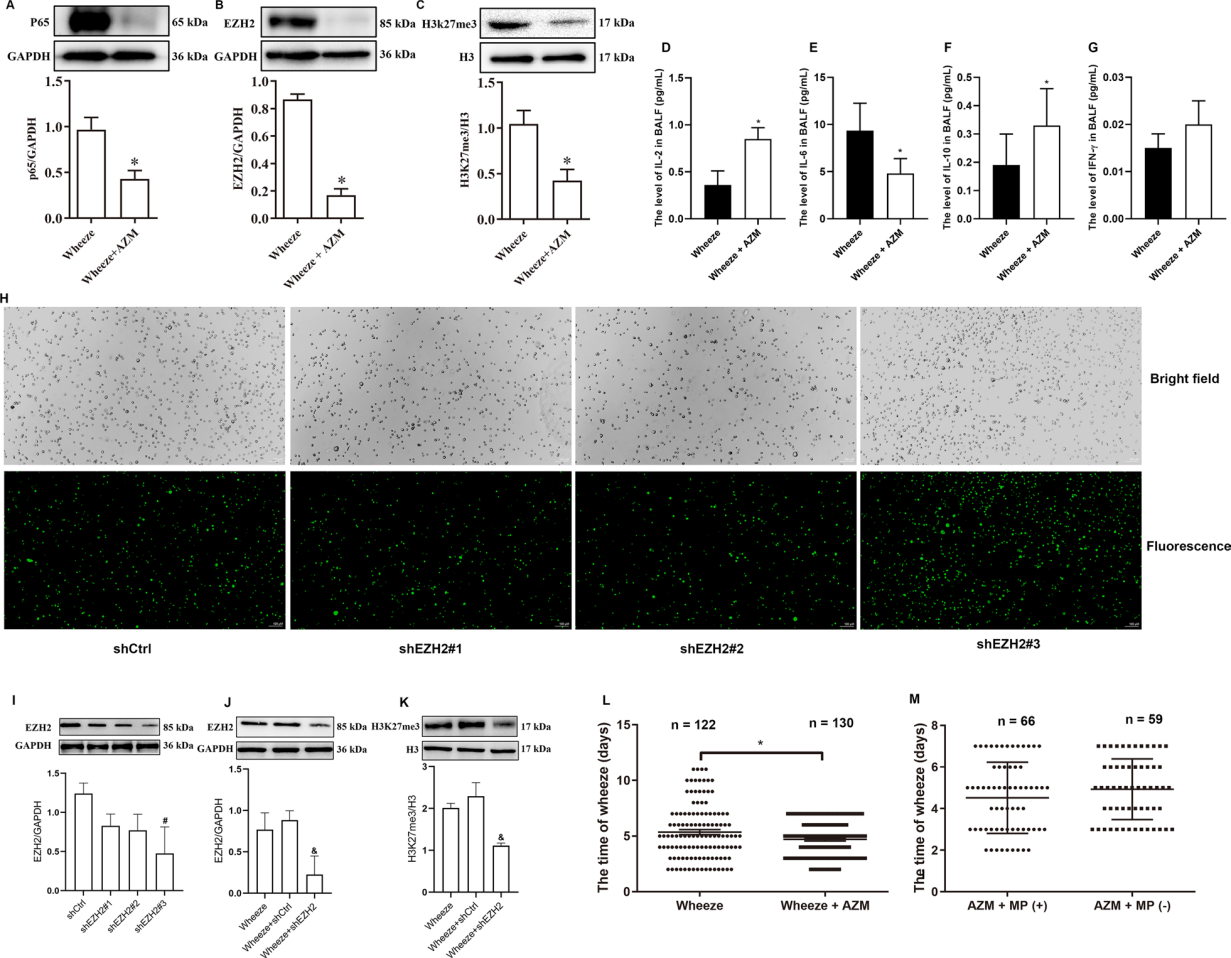
AZM attenuates overexpression of P65, EZH2 and H3K27me3 and cytokines and alleviates wheezing time. **A**–**C** The expression levels of P65, EZH2 and H3K27me3 were examined by Western blotting (*n* = 4). **D**–**G** Effects of AZM on IL-2, IL-6, IL-10 and INF-γ in the BALF (*n* = 3). **H** Three shEZH2 intervention sites were used to optimize the transfection efficiency, and that of the lentiviral vector was analyzed by immunofluorescence containing EZH2 shRNA. Scale bars indicate 100 μm. **I** Lentiviral transfection efficiency of shEZH2 was analyzed by Western blotting. ^#^*P* < 0.05 versus the shCtrl group (*n* = 3). **J**, **K** The expression of EZH2 and H3K27me3 after shEZH2 intervention was detected by protein immunoblotting. ^&^*P* < 0.05 versus the Wheeze + shCtrl group (*n* = 3). **L** Effects of AZM on wheezing time in patients. **M** Effects of mycoplasma infection on AZM and wheezing time. *P < 0.05 versus the Wheeze group

AZM is a macrolide antibiotic and multiple studies have shown that AZM has non-specific anti-inflammatory and immunomodulatory effects, with demonstrated positive effects in a variety of asthmatic diseases(20, 22, 24). Therefore, we examined the anti-inflammatory and immunomodulatory responses in children treated with AZM for 5 days (Wheeze + AZM group) compared with those in children not treated with AZM (Wheeze + Vehicle group). Western blotting results showed that the expression levels of NF-κB P65, EZH2 and H3K27me3 in the Wheeze + AZM group were significantly decreased compared with levels in the Wheeze + Vehicle group (P < 0.05; Fig. 3A–C). These data implied that AZM attenuated the induction of NF-κB P65, EZH2 and H3K27me3 in the BALF of children with wheezing. We further found that IL-2 and IL-10 were increased in the BALF of children with wheezing treated with AZM compared with those in the cases not treated with AZM (P < 0.05; Fig. 3D–F). There was no change in the expression level of INF-γ (Fig. 3G). Furthermore, to verify the role of EZH2-mediated H3K27me3 in lung inflammation in children with wheezing, we further isolated and cultured alveolar macrophages in BALF from children with wheezing and used shRNA to perform EZH2 knockdown of cultured macrophages in vivo. We first used alternative shRNA constructs (shEZH2#1, shEZH2#2, shEZH2#3) targeting different EZH2 sites in macrophages cultured and then evaluated the protein levels of EZH2 by Western blotting. Of the three constructs, more shEZH2#3-positive cells were also detected by immunofluorescence, reflecting the high transfection efficiency of shEZH2#3 (Fig. 3H). The Western blotting results also showed that shEZH2#3 had the strongest inhibition effect on the protein expression of EZH2 (Fig. 3I), verifying the best intervention target for shEZH2. These data indicate that shEZH2#3 has the highest interference efficiency; therefore, we chose shEZH2#3 for EZH2 knockdown of macrophages. Western blotting results after shEZH2 intervention also showed that the expression levels of EZH2 and H3K27me3 in Wheeze + shEZH2 group were significantly lower than those in Wheeze + shCtrl group (Fig. 3J, K). In addition, wheezing time in the Wheeze + AZM group was significantly shortened compared with that in the Wheeze + Vehicle group (*P* < 0.05; Fig. 3L).

AZM is mainly used for the treatment of MP infection. To avoid the impact on any effects caused by MP infection, the Wheeze + AZM group was divided according to the MP infection into the AZM + MP (+) group and AZM + MP (−) group. There was no significant difference in wheezing time between the AZM + MP (+) group and AZM + MP (−) group (Fig. 3M). The above results further implied that AZM may be attenuate wheezing in children through non-specific anti-inflammatory effects but not anti-mycoplasma effects. All in all, our data so far suggested that AZM reduces NF-κB P65, EZH2 and H3K27me3 expression in the BALF of children with post-inflammatory wheezing and shortens their wheezing time.

### LPS stimulation of the rat alveolar macrophage cell line (NR8383) to mimic inflammation and selection of LPS, AZM, GSK126 and SN50 optimal concentrations

The NF-κB signaling pathway is involved in cell response to external stimuli and induces multiple cytokines. Activation of the NF-κB-related inflammatory signaling pathway is closely related to histone H3 methylation modification [16, 27]. To examine whether AZM is involved in histone methylation modification mediated by the NF-κB signaling pathway, we next used LPS to stimulate NR8383 cells to mimic the inflammatory response.

**Fig. 4:**
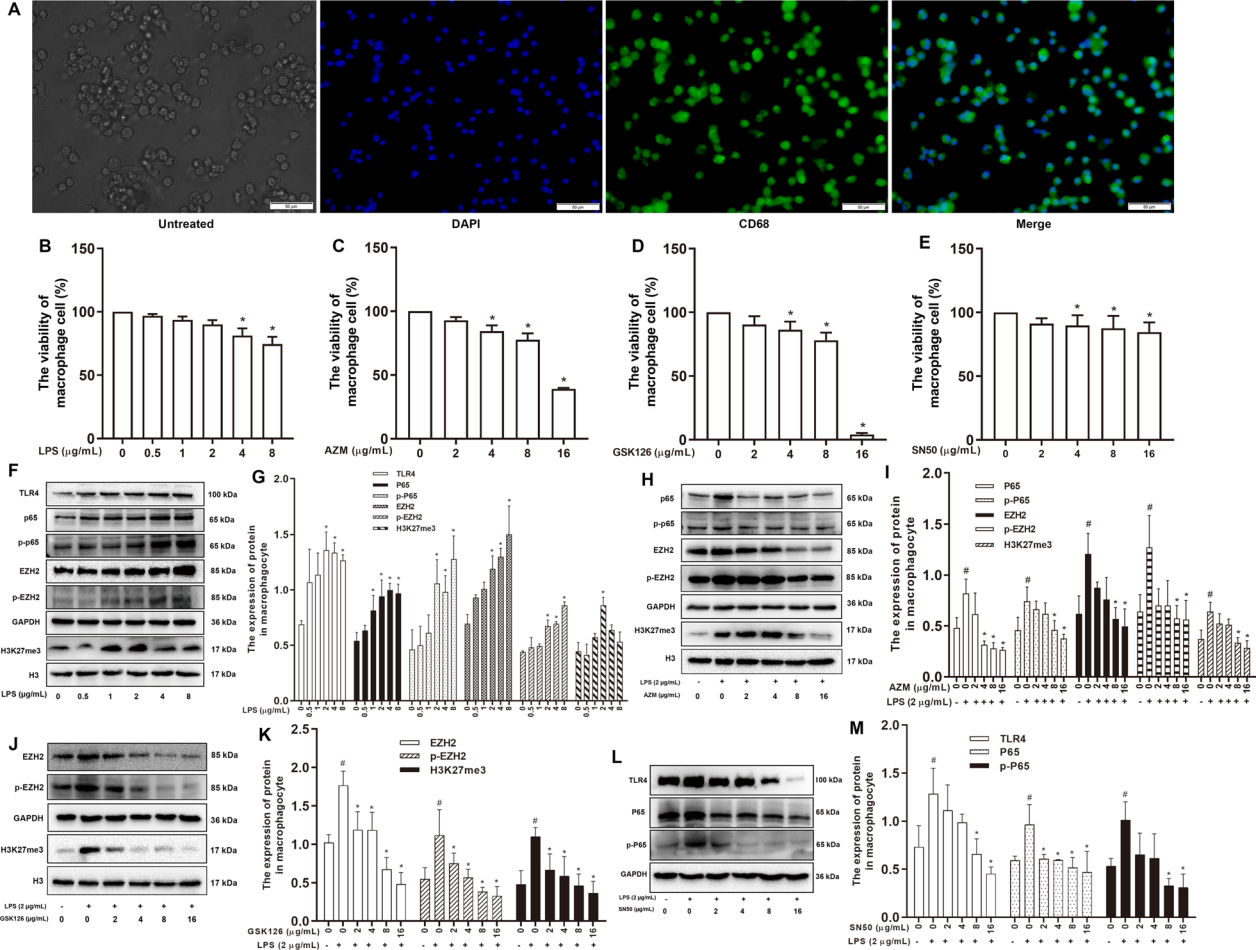
Selection of optimal concentrations of LPS, AZM, GSK126 and SN50 in rat alveolar macrophages. **A** Green (CD68), blue (DAPI). The scale bars represent 50 μm. **B**–**E** The cell viability of different concentrations of LPS, AZM, GSK126 and SN50. *P < 0.05 versus the 0 µg/mL group (*n* = 3). **F** Western blotting analysis of the expression levels of TLR4, NF-κB P65, NF-κB p-P65, EZH2, p-EZH2 and H3K27me3 in rat alveolar macrophages (NR8383) treated with different LPS concentrations and **G** quantification. ^#^*P* < 0.05 versus the 0 µg/mL LPS group (*n* = 3). **H** Western blot analysis of the expression levels of NF-κB P65, NF-κB p-P65, EZH2, p-EZH2 and H3K27me3 in LPS-treated NR8383 cells at different AZM concentrations and **I** quantification. ^#^*P* < 0.05 versus the 0 µg/mL LPS group, *P < 0.05 versus the 2 µg/mL LPS + 0 µg/mL AZM (*n* = 3). **J** Western blotting analysis of the expression levels of EZH2, p-EZH2 and H3K27me3 in LPS-treated NR8383 cells at different GSK126 concentrations and **K** quantification. ^#^*P* < 0.05 versus the 0 µg/mL LPS group, *P < 0.05 versus the 2 µg/mL LPS + 0 µg/mL GSK126 (*n* = 3). **L** Western blotting analysis of the expression levels of TLR4, NF-κB P65 and NF-κB p-P65 in LPS-treated NR8383 cells at different SN50 concentrations and **M** quantification. ^#^*P* < 0.05 versus the 0 µg/mL LPS group, *P < 0.05 versus the 2 µg/mL LPS + 0 µg/mL SN50 (*n* = 3)

NR8383 cells were first identified by immunofluorescence staining for the macrophage-specific antigen CD68 (Fig. 4A). Cell Counting Kit-8 (CCK-8) assay was used to detect the effects of LPS, AZM, GSK126 (an EZH2 inhibitor) and SN50 (a NF-κB inhibitor) at different concentrations on NR8383 cell viability. Compared with cells in the Control group (0 μg/mL), NR8383 cells treated with different concentrations of LPS (0.5, 1, 2, 4, 8 μg/mL), AZM (2, 4, 8, 16 μg/mL), GSK126 (2, 4, 8, 16 μg/mL) and SN50 (2, 4, 8, 16 μg/mL) for 24 h showed a concentration-dependent decrease in cell viability (Fig. 4B–E).

The expression levels of TLR4, P65, p-P65, EZH2, p-EZH2 and H3K27me3 in NR8383 cells after different doses of LPS treatment were detected by Western blotting. The expression levels of all factors in the 2 μg/mL LPS group were higher than that in the control group (0 μg/mL) (*P* < 0.05; Fig. 4F, G). These data showed that 2 μg/mL LPS induced the overexpression of NF-κB P65, EZH2 and H3K27me3 in NR8383 cells. Combined with the effect of LPS on cell viability in Fig. 4B, 2 μg/mL LPS was selected as optimal intervention concentration in NR8383 cells for subsequent experiments.

Next, NR8383 cells with LPS intervention were treated with AZM, GSK126 and SN50, and various concentrations of AZM, GSK126 and SN50 were examined. Western blotting results showed that the expression levels of P65, p-P65, EZH2, p-EZH2, H3K27me3 in the 8 μg/mL and 16 μg/mL AZM groups were significantly decreased compared with those in the LPS group (*P* < 0.05; Fig. 4H, I). Comparing these results with the viability data from Fig. 4C, 8 μg/mL AZM was selected for subsequent experiments. Western blotting results showed that EZH2, p-EZH2 and H3K27me3 levels in the 4 μg/mL GSK126 group were significantly decreased (*P* < 0.05; Fig. 4J, K). Comparing these results with the viability data from Fig. 4D, 4 μg/mL GSK126 was selected for subsequent experiments. TLR4, P65 and p-P65 expressions in the 8 μg/mL SN50 group were significantly decreased compared with levels in the LPS group (*P* < 0.05; Fig. 4L, M). Comparing these results with the viability data from Fig. 4E, 8 μg/mL SN50 was selected for subsequent experiments.

### AZM may regulate histone H3K27me3 methylation modification by inhibiting NF-κB P65 nuclear translocation

**Fig. 5:**
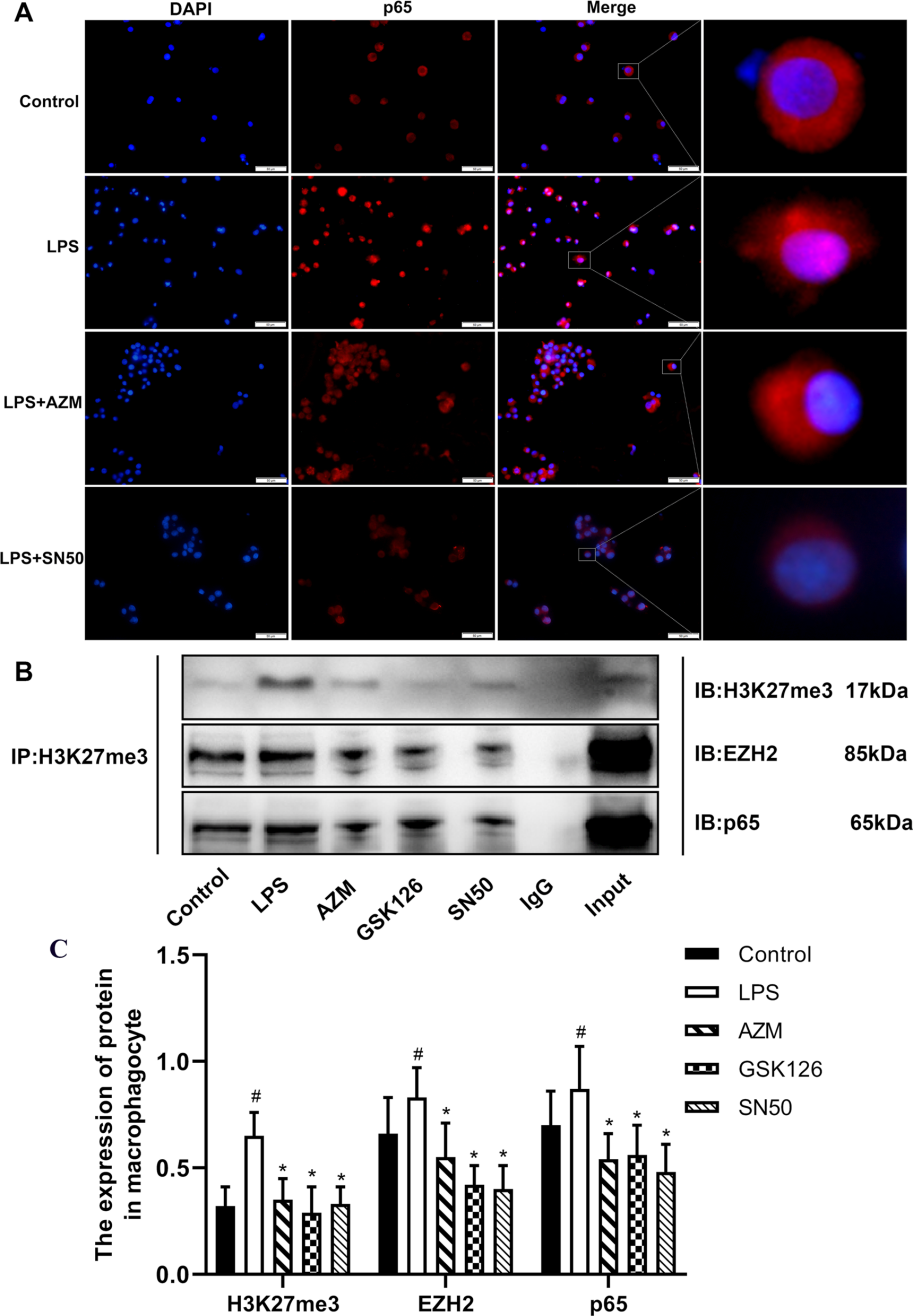
AZM and SN50 inhibit NF-κB P65 nuclear translocation induced by LPS in rat alveolar macrophages. **A** AZM and SN50 suppressed LPS-induced NF-κB P65 nuclear translocation in rat alveolar macrophages (NR8383). Immunofluorescence staining showed NF-κB P65 localization in NR8383 cells. The scale bars represent 50 μm. **B** Interaction of H3K27me3, EZH2 and P65 was detected by Co-IP in NR8383. Co-IP of cell lysates of NR8383 treated as indicated with anti-H3K27me3-protein G magnetic beads and immunoblot (IB) with an anti-EZH2 and anti-NF-κB P65 antibody for evaluation of protein expression. IgG: negative control. **C** Co-IP quantification analysis. ^#^*P* < 0.05 versus the Control group, *P < 0.05 versus the LPS group (*n* = 3)

We next examined the influence of AZM on the NF-κB P65 signaling pathway by examining NF-κB P65 nuclear translocation of LPS-induced NR8383 cells treated with AZM or the NF-κB inhibitor SN50. Immunofluorescence data showed that AZM (8 μg/mL) or SN50 (8 μg/mL) inhibited LPS-induced NF-κB P65 nuclear translocation in NR8383 cells (Fig. 5A).

Macrophages stimulated by LPS activate the NF-κB signaling pathway, which directly up-regulates pro-inflammatory factors and adhesion molecules [28]. Hence, we further detected the relationship between histone H3K27me3, EZH2 and NF-κB P65 in NR8383 cells by Co-IP. The Co-IP results showed that histone H3K27me3 interacted with EZH2 and NF-κB P65 (Fig. 5B, C). These results implied that histone H3K27me3, EZH2 and NF-κB P65 interact with each other.

### AZM normalizes the abnormal expression of IL-10 induced by LPS through inhibiting NF-κB P65-related hypermethylation of H3K27me3

**Fig. 6:**
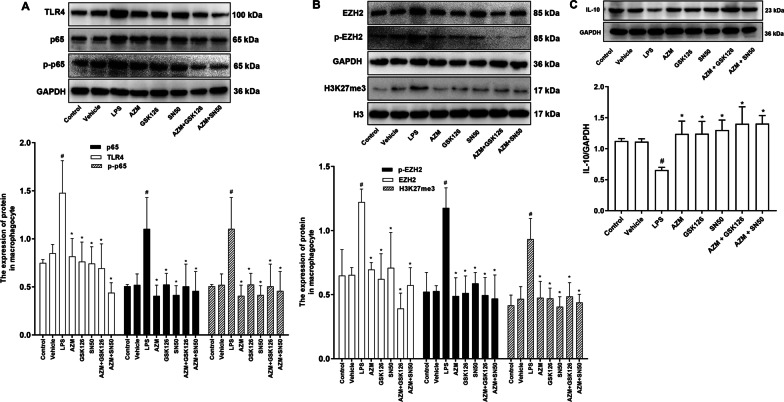
AZM reduces induced IL-10 expression caused by LPS through inhibiting H3K27me3 hypermethylation. **A** Western blot and quantification of the expression levels of TLR4, NF-κB P65 and NF-κB p-P65 in rat alveolar macrophages (NR8383). **B** Western blotting and quantification of EZH2, p-EZH2, H3K27me3 in the NR8383 cells. **C** Western blotting and quantification of the expression levels of IL-10 in the NR8383 cells. ^#^*P* < 0.05 versus the Control group, *P < 0.05 versus the LPS group (*n* = 3)

Inflammation was induced in NR8383 cells by LPS. Cells were treated with AZM, GSK126 and SN50. Western blotting results showed that while TLR4, NF-κB P65, NF-κB p-P65 were increased in LPS-treated cells compared with those in controls, AZM, GSK126 and SN50 attenuated the induction of TLR4, NF-κB P65 and NF-κB p-P65, respectively (*P* < 0.05; Fig. 6A). Cells induced by LPS showed increased expressions of EZH2, p-EZH2 and H3K27me3 compared with controls, and these expressions were significantly decreased in the AZM group, GSK126 group, SN50 group, AZM + GSK126 group and AZM + SN50 group compared with those in the LPS group (*P* < 0.05; Fig. 6B). These results implied that AZM reduces LPS-induced histone H3K27me3 hypermethylation possibly by inhibiting the NF-κB P65 signaling pathway.

Western blotting results showed that IL-10 expression in the LPS group was significantly lower than that in Control group (*P* < 0.05), while IL-10 was significantly increased in the AZM group, GSK126 group, SN50 group, AZM + GSK126 group and AZM + SN50 group (*P* < 0.05; Fig. 6C). These data indicated that AZM restored LPS-mediated reduction of IL-10 possibly by inhibiting NF-κB P65 signaling pathway involved EZH2-mediated hypermethylation of histone H3K27me3 in NR8383 cells.

## Discussion

Multiple studies have shown that while genetic and allergic constitutions can cause asthma, the invasion of respiratory microorganisms and atypical pathogens is the main cause of wheezing and even asthma [2, 29]. Macrophages, a type of immune cell, have dual effects of both anti-inflammation and pro-inflammation, and dysfunction of macrophages may lead to the development of asthmatic diseases. Macrophages account for more than 80% of the cells in BALF in lung inflammatory diseases [30]. Activated macrophages induce multiple cytokines to eliminate external invasive substances and help restore homeostasis. IL-6, IL-1 and TNF-α are highly expressed in the BALF of asthma patients; these cytokines induce airway inflammatory cell infiltration and airway injury and aggravate bronchial hyperresponsiveness [31]. Clinical studies have shown that children with recurrent wheezing and poor conventional medical treatment show significantly alleviated wheezing after bronchoscopy lavage, indicating that inflammatory factors play an indispensable role in wheezing after pulmonary inflammation. Our study found that IL-6 and IL-2 were increased significantly, while IL-10 was decreased in the Wheeze group, indicating that anti-(pro-)inflammatory factors imbalance may play a critical role in the occurrence and development of wheezing after infection. However, in our study, the expression of IL-2 was apparently increased, while IL-10 was decreased in Non-wheezing group when compared to the Control group. Pulmonary infections are also present in Non-wheezing patients, so anti-(pro-)inflammatory factors in BALF of Non-wheezing group may also increase or decrease, but the extent of cytokines change in the Wheezing group was higher than that in the Non-wheezing group. The above results further suggested that the occurrence of wheezing after infection may be related to the degree of cytokines change in the BALF. IL-6 is a cytokine with the fastest response to injury and infection and has high diagnostic value in the early stage of infection. IL-6 shows pro-inflammatory effects and is mainly produced by macrophages, which can be induced in the early response to inflammation [32]. IL-2 is mainly produced by the activation of T lymphocytes and plays an important role in immune response and anti-infection. IL-2 induces the proliferation of T lymphocytes stimulated by specific antigens or mitogenic factors, promotes the proliferation and differentiation of B lymphocytes, generates antibodies and activates macrophages [33]. IL-10, an anti-inflammatory factor, antagonizes the pro-inflammatory function of other biological factors and the aggregation of adhesion factors to inhibit inflammation. IL-10-producing Treg cells inhibit Th2 responses, such as IgE conversion, eosinophilia and antihyaluronidase reaction response to antigen, thereby inhibiting the inflammatory response and reducing airway epithelial shedding [34]. A pro-inflammatory response and insufficient anti-inflammatory response can cause various clinical symptoms of immune homeostasis imbalance.

Epigenetics, as the central link between genes and the environment, is involved in the pathophysiological responses of various diseases. Epigenetic studies on asthma have mainly focused on DNA methylation. Notably, DNA methylation can affect histone methylation and vice versa [35]. After LPS stimulation of macrophages, the NF-κB signaling pathway is activated. NF-κB directly up-regulates pro-inflammatory factors and adhesion molecules and activates the transcription of the demethylase JMJD3 [28]. Increased JMJD3 activity resulted in decreased methylation of H3K27 in the promoter of M2 macrophage-related genes [36]. These studies suggested that histone H3K27 methylation modification is closely related to the expression of inflammatory factors after infection. EZH2 plays an important role in gene repression by catalyzing histone H3K27me3 to compact chromatin. EZH2 is a main regulator of cell cycle progression, autophagy and apoptosis, promoting DNA damage repair, inhibiting cell senescence and participating in drug resistance [37]. In the present study, immunofluorescence results showed that the expression level of EZH2 and histone H3K27me3 in the Wheeze group was higher than that in the Control group. Whether this is the direct cause of the decrease in the anti-inflammatory factor IL-10 and the occurrence of post-pneumonic wheezing is unclear and will be the subject of future investigations.

AZM has been widely recognized as an effective treatment for wheezing diseases [38, 39]. In this study, in order to further explore the role of AZM on wheezing after infection, wheezing children were treated with AZM, and the wheezing time was used to evaluate the effect of AZM on wheezing after infection in children. We found that the wheezing time was significantly shortened after AZM treatment, which confirmed the application value of AZM in wheezing diseases in children. We also examined the effects of MP infection on AZM treatment. The patients with wheezing treated with AZM were divided into two groups on the basis of MP infection; however, there was no significant correlation between MP infection and the efficacy of AZM in the treatment of wheezing diseases, which implied that AZM may be attenuate wheezing in children through non-specific anti-inflammatory effects but not anti-mycoplasma effects. Some studies have suggested that AZM reduces the expression of a variety of cytokines, such as TRAF6 and IL-6 [40, 41]. Therefore, we further examined the effects of AZM on inflammatory factors in the BALF, and the data showed that AZM not only decreased the expression level of the pro-inflammatory factor IL-6, but it also promoted the expression level of anti-inflammatory factor IL-10. This showed that AZM could restore the imbalance of pro-inflammatory and anti-inflammatory factors in the BALF. IL-10, also known as human cytokine synthesis inhibitor, is an anti-inflammatory cytokine that mainly plays the role of down-regulating inflammatory response and antagonizing inflammatory mediators. More importantly, IL-10 can inhibit the specific immune function of macrophages, enhance immune induction, inhibit the release of inflammatory factors by macrophages and reduce the expression of adhesion molecules, so that IL-10 plays an important role in the immune mediating of human body [34, 42, 43]. In this study, IL-10 was significantly reduced in BALF of children with wheezing, while it was significantly increased after the treatment of AZM. These findings suggested that IL-10 may play a positive regulatory role in children with wheezing diseases, which is beneficial to the recovery of wheezing.

Noteworthily, in this study, IL-2 increased in BALF of children in the wheezing group, and the increase was more obvious after the treatment of AZM. Many studies have confirmed that IL-2 has antiviral, anti-tumor and immune-enhancing effects [44, 45] and can also play a pro-inflammatory role under certain conditions [46]. It is implied that IL-2 is an adaptive cytokine that can exert both anti-inflammatory and pro-inflammatory effects. Thus, we speculated that IL-2 may play a positive role in immune regulation and inhibition of inflammation in wheezing diseases, which is beneficial to the recovery of wheezing. Additionally, IL-2 is secreted by lymphocytes, while AZM is mainly aggregated in macrophages [47], which may be one of the cause of the increase in IL-2 after AZM treatment in this study. However, the specific mechanism of IL-2 increase in the BALF of wheezing children is not entirely clear. Furthermore, our data of western blotting showed that AZM reduced the expression levels of NF-κB P65, EZH2 and H3K27me3 in the BALF in children with wheezing after pulmonary infection. We speculated that AZM may play a non-specific anti-inflammatory role by regulating histone H3K27me3 methylation mediated by EZH2 and NF-κB P65.

To examine this possibility, we established an inflammation model using LPS in rat alveolar macrophages. LPS, as a typical ligand of TLR4, is recognized by TLR4 and activates intracellular signaling pathways in the form of the LPS-CD14-MD-2 complex [48]. The NF-κB signaling pathway is a vital inflammatory pathway in the body that stimulates the production of cytokines. Our research results showed that the expression levels of TLR4, NF-κB P65 and NF-κB p-P65 increased after LPS treatment in rat alveolar macrophages, which was consistent with the literature [49]. The histone Lys demethylase KDM3C has anti-inflammatory effects by inhibiting NF-κB signal transduction [16]. This also suggests that histone methylation modification is involved in NF-κB signaling pathway activation. In our study, we confirmed an interaction among EZH2, H3K27me3 and NF-κB p65 by Co-IP. GSK126, an inhibitor of EZH2, downregulates pro-inflammatory cytokines through decreasing histone H3K27me3 methylation [50]. After LPS intervention, EZH2 and H3K27me3 were highly expressed in rat alveolar macrophages, while IL-10 was expressed at low levels, so we speculated whether it could inhibit the secretion of IL-10. One report showed that activation of the CD4OL-CD40 signaling pathway in the inflammation of pulmonary adventitial fibroblasts up-regulates YY-1 protein expression, increasing the binding of YY-1 and EZH2 to the promoter region of IL-10 and enhancing histone H3K27me3 levels, leading to inhibition of IL-10 activation; these results confirmed that H3K27me3 plays a key role in the negative transcriptional regulation of IL-10 [51]. In this study, we did not directly verify whether the promoter region of the IL-10 gene was regulated by histone H3K27me3, which was a limitation of our study.

AZM is a commonly used antibiotic, with high safety, low adverse reactions in children and low liver toxicity. AZM accumulates in lung tissue and leads to increased macrophages at the lesion sites [47]. After lung injury, epithelial cells produce large amounts of transforming growth factor-β1, inflammatory cells and fibroblasts to induce persistent and severe subcutaneous fibrosis. Studies showed that AZM can reverse this process [52]. AZM has immunoregulatory effects on severe asthma, reduces the levels of interleukin and TNF-α and prevents airway remodeling [53]. A recent report showed that the long-term low-dose use of AZM inhibits the production of pro-inflammatory cytokines and enhances macrophage phagocytosis and anti-inflammatory cytokine expression [54]. Studies have also found that AZM may be protective against wheezing in acute bronchiolitis and that AZM treatment reduces the need for short-acting β2-agonists in preschool children with recurrent wheezing [55]. These data suggested that AZM has a positive effect on wheezing diseases after pulmonary inflammation. Here, we explored whether AZM exhibits its functions through epigenetic regulation. In LPS-stimulated rat alveolar macrophages, AZM reduced the induction of NF-κB p65, EZH2 and histone H3K27me3 levels. We further found that AZM inhibited the nuclear translocation of NF-κB p65 and promoted the expression of IL-10. Together, these data suggested that AZM might transcriptionally increase IL-10 expression by inhibiting EZH2-mediated histone H3K27me3 hypermethylation.

## Conclusion

**Fig. 7:**
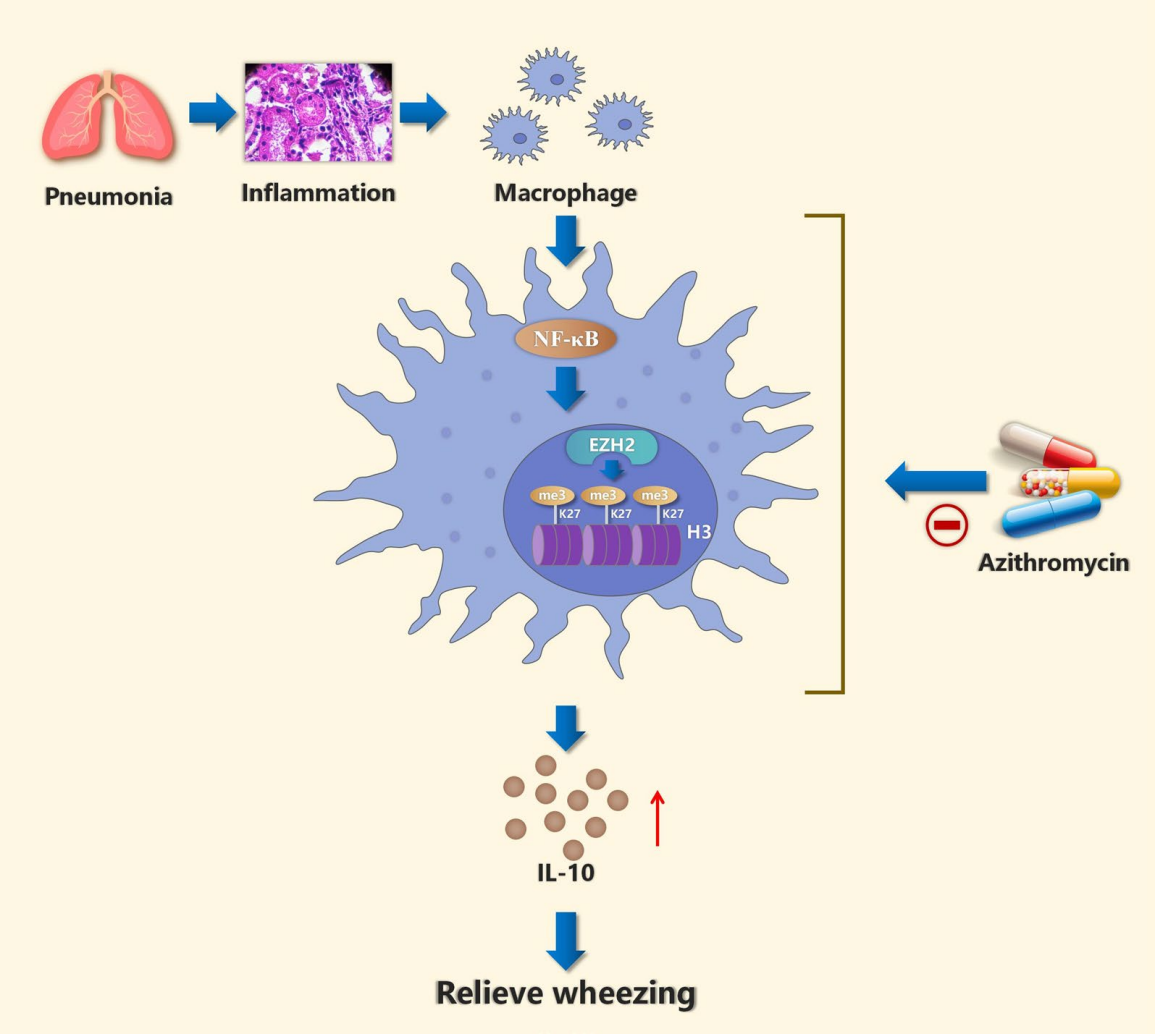
Schematic representation of the potential mechanism by which AZM attenuates post-inflammation wheezing. Abnormal expression of IL-10 was observed in children with wheezing after pulmonary inflammation. AZM could normalize the abnormal expression of IL-10 through inhibiting NF-κB P65-related histone H3K27me3 hypermethylation induced by EZH2

Our results suggest that AZM up-regulates the expression of IL-10 in the BALF of patients with wheezing by inhibiting the NF-κB signaling pathway and down-regulating the EZH2-mediated histone H3K27me3 hypermethylation (Fig. 7). These findings may assist in the development of novel methods for preventing and treating wheezing caused by pulmonary inflammation diseases in children.

## Materials and methods

### Human subjects and specimens

BALF samples were collected from children aged 6 months to 12 years at the Affiliated Hospital of Zunyi Medical University from January 1, 2021, to December 1, 2021. All children were indicated for fiber-optic bronchoscopy. A fiber-optic bronchoscope (BF-1T260, Olympus, Tokyo Japan) was used. The methods were carried out in accordance with the approved guidelines [56]. The study was approved by the hospital ethics committee (KLL-2021-059), and the guardians of patients provided written informed consent in accordance with the Declaration of Helsinki (revised in 2013). Subjects were divided into the Non-wheeze group (diagnosed with severe pneumonia without wheezing) (72 cases) and Wheeze group (diagnosed with severe pneumonia with wheezing) (132 cases). The Control group included 23 cases diagnosed with bronchial foreign body in acute stage, congenital laryngeal cartilage dysplasia or airway malformation; no patient showed airway infection.

The Wheeze group was further divided into two subgroups: Wheeze + Vehicle group (intravenous infusion of 5% glucose without AZM) (76 cases) and Wheeze + AZM group (5% glucose injection containing AZM given intravenously at a dose of 10 mg/kg·d, 5 days of continuous use [57]) (56 cases). In addition, all patients routinely received third-generation cephalosporin anti-infective and symptomatic treatment. The Wheeze + AZM cases were further subdivided depending on infection with MP: the AZM + MP positive group (serum mycoplasma antibody > 1:160 positive or BALF mycoplasma RNA positive) and the AZM + MP negative group. When the children’s lungs hear wheezing rale, the time of wheezing was counted, and wheezing time was evaluated as the length of time it took for wheezing in the lungs to go away. The 2011 clinical practice guidelines for community-acquired pneumonia in children in the USA were used in the study [58].

BALF was collected after 5–7 days of treatment and centrifuged at 2000×*g* for 10 min at 4 °C. The supernatant and precipitation were stored at − 80 °C for further analysis.

### Isolation and culture of alveolar macrophages (AMs) in vivo

The recovered BALF was filtered through 2-layer sterile gauze and centrifuged at 500×*g* for 10 min at 4 °C. The cell pellets were suspended with PBS and washed three times and suspended with RPMI-1640 (Thermo Fisher Scientific, USA) containing 10% heat-inactivated fetal bovine serum (MRC, China), 100 U/mL penicillin and 0.1 mg/mL streptomycin (Solarbio, Beijing, China). The cell suspension was added at 0.5 × 10^6^ cells/well to a 24-well plastic tissue culture plate and incubated at 37 °C in a 5% CO_2_ humidified milieu for 2 h and then washed with PBS three times to remove the adherent cells and obtain adherent AMs.

### Short hairpin RNA (shRNA) and retroviral infections

One hundred nanograms of EZH2‐specific shRNA was used. The lentivirus promoter driving the expression of shRNA and the shRNA sequence was inserted. The expression of the reporter, enhanced green fluorescent protein (eGFP), was driven by the Ubi promoter. shRNA and eGFP sequences were incorporated into a lentivirus. Lentiviruses were produced in 293 T cells, and viral titers of 2 × 10^9^ TU/mL were used. The cells were seeded on a 6‐well culture plate at 1 day before infection. Fresh medium containing 5 µg/mL polybrene and the virus (MOI = 4) were added to the cells. Fluorescence signals were observed under a fluorescence microscope after 72 h of cell infection.

### Cell culture and treatment

The rat alveolar macrophage cell line NR8383 (purchased from National Collection of Authenticated Cell Cultures, CSTR:19375.09.3101RATGNR9) was cultured in Ham’s F12K medium supplemented with 2 mM L-glutamine, 1.5 g/L sodium bicarbonate and 20% fetal bovine serum under a humidified atmosphere of 5% CO_2_ at 37 °C.

The following reagents were used in some experiments: LPS (Sigma-Aldrich, St. Louis, MI, USA), AZM (HIDRAGON, HuBei, China), GSK126 (MCE, Shanghai, China) and SN50 (MCE).

In some experiments, cells were divided into groups: Control group; Vehicle group (DMSO); LPS group (LPS 2 µg/mL); AZM group (LPS 2 µg/mL + AZM 8 µg/mL); GSK126 group (LPS 2 µg/mL + GSK126 4 µg/mL); and SN50 group (LPS 2 µg/mL + SN50 8 µg/mL). To evaluate the additive inhibitory effect of AZM and two inhibitors (GSK126, SN50), we performed cell experiments using the AZM + GSK126 group (LPS 2 µg/mL + AZM 8 µg/mL + GSK126 4 µg/mL) and AZM + SN50 group (LPS 2 µg/mL + AZM 8 µg/mL + SN50 8 µg/mL). Cells were treated as indicated for 24 h and then analyzed.

### Cell viability assay

Cell Counting Kit-8 (Solarbio, Beijing, China) assay was used to examine cell viability. NR8383 cells were prepared as a single cell suspension and inoculated into 96-well plates (100 µL per well) in duplicate wells and cultured for 24 h at 37 °C, 5% CO2. Cells were then treated with LPS (0, 0.5, 1, 2, 4, 8 µg/mL), AZM (0, 2, 4, 8, 16 µg/mL), GSK126 (0, 2, 4, 8, 16 µg/mL) or SN50 (0, 2, 4, 8, 16 µg/mL) for 24 h. Finally, 10 μL CCK-8 solution was added and the cells were placed in an incubator for 2 h in the dark. The absorbance value at 450 nm was determined.

### Immunofluorescence

Cells were resuspended in medium Ham’s F12K containing 20% fetal bovine serum (FBS) and cultured on Thermanox plastic coverslips (Thermo Fisher Scientific, USA) for 24 h. After fixation with 4% paraformaldehyde, the cells were treated with 0.5% Triton X-100 in PBS and 10% normal goat serum and then incubated with primary antibody (anti-EZH2 [1:200, Proteintech], anti-CD68 [1:3000, Proteintech], anti-H3K27me3 [1:1000, Abcam] or anti-P65 [1:200, Proteintech]) at 4 °C overnight. After washing, Alexa Fluor 488 goat anti-mouse IgG secondary antibody and Alexa Fluor 594 goat anti-mouse IgG secondary antibody (1:1000, Thermo Fisher Scientific) were added and samples were incubated in the dark for 1 h at 37 °C. Cells were washed with PBS and counterstained with DAPI. Finally, cells were treated with anti-quench sealing agent and observed under a fluorescence microscope. Fluorescence quantification was performed using ImageJ software (Version 1.47, NIH, USA).

### Western blotting analysis

Cells were collected, and proteins were extracted using the Nuclear Protein Extraction Kit (Merck Millipore, Darmstadt, Germany); the protein concentrations of lysates were determined using the BCA Protein Assay Kit (Solarbio, Beijing, China). Equal quantities of proteins were separated by 8%-15% sodium dodecyl sulfate–polyacrylamide gel electrophoresis and transferred to polyvinylidene difluoride membranes. The membranes were blocked with 5% non-fat milk and then incubated with the following primary antibodies overnight at 4 °C: TLR4 (1:2000, Proteintech), EZH2 (1:2000, Proteintech), p-EZH2 (1:1000, Proteintech), P65 (1:2000, Proteintech), p-P65 (1:1000, Abcam), H3K27me3 (1:1000, Abcam) and IL-10 (1:3000, Proteintech). Membranes were then incubated with the corresponding horseradish peroxidase-conjugated secondary antibodies (1:5000, Proteintech) for 1 h at 4 °C, and protein bands were visualized by enhanced chemiluminescence (Thermo Fisher Scientific, USA). Band intensity was quantified using ImageJ software (Version1.47, NIH, USA).

### ELISA

ELISA was used to detect the levels of interleukin-2 (IL-2), interleukin-6 (IL-6), interferon-gamma (IFN-γ) and interleukin-10 (IL-10) in BALF. ELISA kits (MultiScience, China) were placed at room temperature (RT) for equilibrium, and BALF samples were brought to RT to avoid repeated freezing and thawing. Standard dilution reagent (230 μL) was added to each EP to generate a dilution gradient, and a standard curve was prepared. The appropriate antibody was added, and plates were incubated at RT for 2 h. The liquid was discarded, and the plates were spin dried. Each well was washed with 300 μL of 1 × washing buffer for 2 min, and the buffer was removed. Next, 100 μL of streptavidin-HRP was added to each well and samples were incubated at RT for 30 min. After washing, 100 μL of TMB substrate solution was added at RT for 30 min. Stop solution was added to each well, and a microplate reader (Bio-Rad, USA) was used to read samples at 450 nm.

### Total RNA extraction and real-time quantitative polymerase chain reaction (RT-qPCR)

Total RNA was extracted from the collected BALF using an RNA extraction kit (BioTeke, Beijing, China) as per the manufacturer’s protocol. The RNA was then reverse-transcribed into a single-stranded cDNA using the AMV Reverse Transcription System (Takara, Dalian, Liaoning, China). Then, cDNA was amplified with SYBR Green dye kit and gene-specific primer (Takara, Shiga, Japan). The data were normalized to *β-actin* mRNA levels. Relative gene expression was measured by 2^−ΔΔCt^ method. Primer sequence design is as follows:

- *EZH2* (F): AGTTCGTGCCCTTGTGTGATAGC
- *EZH2* (R): ACTCTCGGACAGCCAGGTAGC
- *β-actin* (F): GGCCAACCGCGAGAAGATGAC
- *β-actin* (R): GGATAGCACAGCCTGGATAGCAAC.

### Co-immunoprecipitation (Co-IP)

Co-IP assays were performed using a BeaverBeads™ Protein A/G Assay kit (Beaver, China). NR8383 cells (1 × 10^6^) were lysed in 300 μL IP binding buffer with 1 mM PMSF protease inhibitor on ice for 30 min. Lysates were centrifuged at 17,000 g for 10 min at 4 °C. One-quarter of the supernatant was held as input, and the remaining supernatant was used for the Co-IP assay. Protein A/G agarose beads (50 μL) washed with 200 µL binding buffer were added to 200 μL of lysate and incubated with anti-IgG (1:500, Cell Signaling Technology, Inc.) or anti-H3K27me3 (1:250, Abcam) overnight at 4 °C with slow shaking. The sample was washed with 200 µL washing buffer, and beads were magnetically separated. The immobilized protein complex was eluted at 95 °C in 5X SDS-PAGE Loading Buffer (20 μL) for 10 min. The supernatant was removed, and Western blotting was performed using anti-H3K27me3 (1:1000, Abcam), anti-EZH2 (1:2000, Proteintech) and anti-P65 (1:2000, Proteintech). IgG was used as a negative control.

### Statistical analysis

SPSS statistical software package version 18.0 (SPSS Inc, Chicago, IL, USA) was used for statistical analysis. Statistical analysis was performed using t test or one-way analysis of variance with post hoc comparisons using the least significant differences *t* test. *P* values < 0.05 were considered statistically significant. All data in this study are given as mean ± standard deviation.

## Data Availability

All data generated or analyzed during this study are included in this published article and its supplementary information files.

## Abbreviations

AZM: Azithromycin
NR8383: The rat alveolar macrophage cell line
BALF: Bronchoalveolar lavage fluid
Co-IP: Co-immunoprecipitation
EZH2: Enhancer of zeste homolog 2
H3K27me3: Histone H3 trimethylated at lysine 27
IFN-γ: Interferon-gamma γ
TNF: Tumor necrosis factor
IL-1: Interleukin-1
IL-2: Interleukin-2
IL-6: Interleukin-6
IL-10: Interleukin-10
LPS: Lipopolysaccharide
MP: Mycoplasma
NF-κB: Nuclear factor kappa-B
SN50: NF-κB inhibitor
GSK126: EZH2 inhibitor
TLR4: Toll-like receptor 4
RT: Temperature
